# Dendritic Cell Status Modulates the Outcome of HIV-Related B Cell Disease Progression

**DOI:** 10.1371/journal.ppat.1002154

**Published:** 2011-08-25

**Authors:** Johanne Poudrier, Michel Roger

**Affiliations:** 1 Laboratoire d'immunogénétique, Centre de Recherche du Centre Hospitalier de l'Université de Montréal (CHUM), Montréal, Québec, Canada; 2 Département de Microbiologie et Immunologie de l'Université de Montréal, Montréal, Québec, Canada; The Fox Chase Cancer Center, United States of America

The overall outcome of HIV disease may depend on the host's capacity to maintain dendritic cell (DC) homeostasis at mucosal sites, where DC populations, one of the earliest cell types to be exposed to the virus, present an inherent capacity to modulate the balance between tolerance and protection. DCs may influence mucosal B cell responses against HIV through contact and/or production of B cell growth factors such as B lymphocyte stimulator (BLyS/BAFF), which in turn modulate the outcome of CD4[superscript]+[/superscript] T cell HIV effectors/targets. Recent observations of HIV/SIV infections in non-pathogenic animal models and from mucosal vaccination of nonhuman primates suggest that maintenance of systemic integrity may be achieved through constraining highly efficient immune responses to mucosal sites.

## Do Dendritic Cells Drive B Cell Dysregulation in the Context of HIV Disease Progression?

B lymphocyte disorders are important consequences of HIV infection (reviewed in [1]) and can persist despite therapy and in the absence of apparent disease progression [2]–[5]. DCs play a pivotal role in regulating the outcome of B cell development, activation, and survival. This is mediated mainly through production of B cell growth factors such as BLyS/BAFF [6]–[8]. It is therefore likely that DC alterations associated with HIV infection [9] have an effect on the B cell compartment. Early data supporting this hypothesis were obtained with HIV-transgenic mice, which develop a Nef-dependent AIDS-like disease [10]. In these animals, myeloid DCs (mDCs) present an immature phenotype and altered stimulatory capacities. They accumulate in the enlarged splenic marginal zone (MZ), likely contributing to polyclonal B cell activation and disruption of tolerance [11], [12]. BLyS over expressing mice also present enlarged splenic MZ, B cell hyperactivity, and autoimmunity [13]. This phenotype is also shared by autoimmune-regulatory (AIRE)-deficient mice, in which bone marrow–derived DCs over express BLyS [14], [15]. Interestingly, AIRE is involved in regulation of STAT1 signalling, a pathway also used by HIV-Nef to promote pro-inflammatory monocytes in humans [16], [17] and likely over expression of tumour necrosis factor (TNF)-α by DCs [18]. HIV-gp120 can also mediate B cell activation. Indeed, the binding of gp120 to mannose C-type lectin receptors on B cells up-regulates the class switch recombination (CSR)-inducing enzyme, activation-induced cytidine deaminase, resulting in immunoglobulin (Ig) class switch from IgM to IgG and IgA with the help of BLyS [19]. Furthermore, signalling through toll-like receptor (TLR)7, which binds to HIV-ssRNA, up-regulates BLyS expression in DCs [20]. This, along with the fact that TLR7 is over expressed in blood DCs from individuals with primary HIV infection [21], further suggest that excessive BLyS production by DCs may be involved in triggering and driving B cell dysregulation in the context of HIV.

In recent longitudinal studies involving individuals with HIV with different rates of disease progression, we have shown that mDC levels were reduced in the blood of rapid and classic progressors, beginning in the acute phase of infection and persisting throughout the course of disease despite successful therapy [22]. This correlated with increased serum levels of DC-tropic chemokines, suggesting drainage to peripheral sites [23]. Most importantly, HIV progressors had increased levels of BLyS expression in the plasma and on the surface of both mature blood mDCs and CD11c^+^CD14^+^CD16^−^ monocytic DC-precursors [2]; the latter have been shown to be associated with inflammatory conditions [24]. In these subjects, B cell dysregulation was found throughout disease progression and was accompanied by the increased frequency of a population presenting features shared by both transitional immature (TI) and MZ B cells [2]. These cells express low levels of CD21, suggestive of a non-resting state, and we have thus named this population “precursor/activated MZ-like” B cells. Although human MZ B cells share many common properties with their rodent counterparts, they are not restricted to the spleen. MZ-like B cells re-circulate in humans, and have been identified in several lymphoid tissues such as the inner wall of the sub-capsular sinus of lymph nodes, in the crypt epithelium of tonsils, and under the dome epithelium of Peyer's patches in gut-associated lymphoid tissues (GALT) [25]. However, the human MZ is a complex heterogeneous niche, and therefore further characterization is required to identify the exact nature of the “precursor/activated MZ-like” B cells. Nevertheless, we think that these cells represent a “first line” B cell population that increases in the context of inflammatory conditions such as in HIV infection. Indeed, TI B cells have been found to be elevated [26] and to preferentially give rise to MZ type B cells under conditions of lymphopenia associated with pathology [27]. The fact that TI B cells are hyper-responsive to BLyS [28] and are increased in the blood of HIV-infected patients with advanced disease [29] suggests that BLyS over expression may contribute to increased survival of TI B cells and favoured selection into a MZ-like first line B cell pool [30]. Given the location of first line B cells in lymphoid organs and mucosal-associated lymphoid tissues (MALT), these cells are highly influenced by DCs and constitute a T cell–independent defence against invading pathogens [31]. Also, given their frequent auto-reactive and cross-reactive repertoires and their relative hyperactivity, these populations are often found in pathologic conditions associated with infection, autoimmunity, and lymphomas [28], [31]. The aberrant expression of BLyS and/or its receptors is often linked to B cell autoimmunity and malignancies, favouring the survival and emergence of self-reactive cells at the TI stage [28], [32], [33]. Recently, elevated expression of BLyS was found to be associated with the expansion of TI and MZ-like B cells in salivary glands of patients suffering from Sjögren's syndrome [28]. A similar phenomenon most likely occurs during HIV infection, as supported by the correlation between elevated blood levels of auto-antibodies and high levels of BLyS expression in the plasma and on the surface of blood monocytes of individuals with HIV [34], [35]. Thus in the context of HIV disease progression, there appears to be an early commitment to produce “inflammatory” DCs expressing high levels of BLyS that are recruited to the periphery, where they contribute to B cell dysregulation. This phenomenon seems to affect mainly immature and first line populations, allowing for emergence of a disturbed and self-reactive repertoire that can lead to autoimmune manifestations and malignancies. However, whether this process is regulated by the host response and/or modulated by direct and indirect viral effects remains to be established.

## Control of HIV Disease Is Associated with Unaltered DC Status and Preservation of the B Cell Compartment

In contrast to observations in rapid and classic HIV progressors, blood mDCs and BLyS levels remained unaltered in aviremic slow progressors or “elite controllers” [2], [22]. However, monocytic DC-precursors of a CD11c^+^CD14^+^CD16^+^ phenotype, which murine analogs settle peripheral organs in steady state conditions [24], were found to be significantly increased in the blood of elite controllers [22], suggesting high turnover in the absence of inflammation. Although the percentage of circulating activated mature B cells and precursor/activated MZ-like B cells remained unaltered in elite controllers, the proportion representing a population with features of unactivated “mature MZ” B cells was lower in these individuals when compared to both classic and rapid HIV progressors as well as healthy donors [2]. Although this may reflect early stages of malfunction, we rather favour the view that the capacity to recruit this population to peripheral sites may be beneficial to the “control” of disease progression.

**Figure 1:**
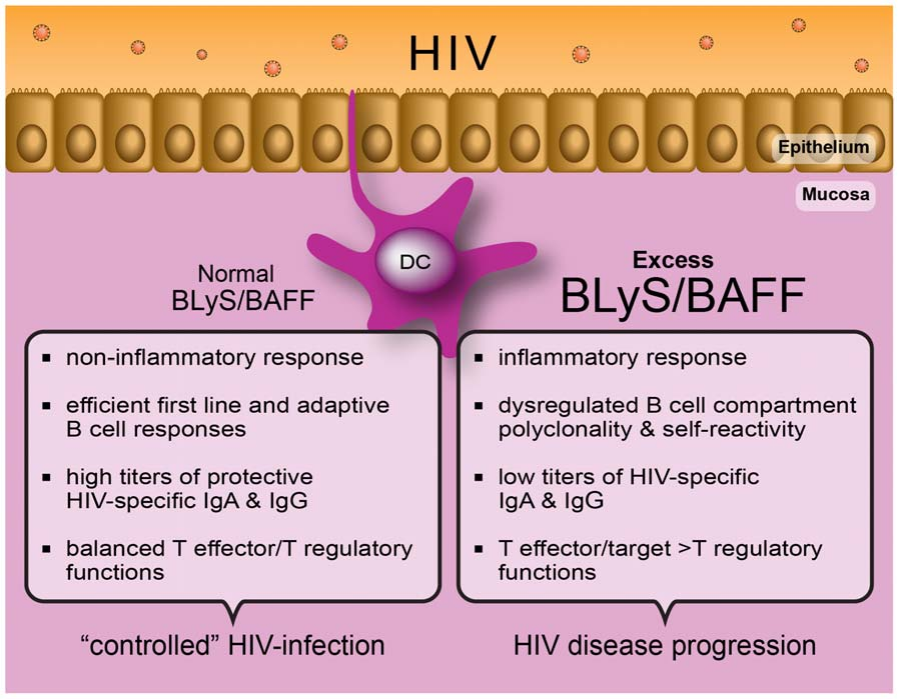
The capacity to control immune homeostasis at mucosal sites, where the main battle against HIV takes place, is reflected by a normal “non-inflammatory” BLyS/BAFF expression status. This is likely modulated through efficient epithelial cell:DC cross talk, subsequently allowing for the generation of highly protective HIV-specific B and T cell responses. In contrast, establishment of an imbalance at the level of mucosal immune homeostasis will allow the excess “inflammatory” BLyS/BAFF expression status to lead to dysregulated B and T cell responses, impairing the generation of highly protective HIV-specific immunity. (Graphic art: Christian Charbonneau.)

Given that mucosal DC populations are gatekeepers of peripheral integrity and amongst the first to be involved in the battle against HIV, it is likely that they influence the outcome of mucosal B cell responses towards the virus [8], [31], [36]. Mucosal HIV-specific IgA are abundant in highly exposed persistently seronegative (HEPS) individuals [37], but rather low in the context of HIV disease progression [1]. Although the issue of “protection” conferred by mucosal HIV-specific IgA remains controversial [37], in many studies these antibodies have been found to neutralize infection and inhibit viral transcytosis in vitro. Furthermore, HIV-gp41 specific mucosal IgA produced by cervical B cells from HEPS individuals presented signs of hypermutation and affinity maturation [38]. Together, these observations based on natural control/immunity versus HIV suggest that efforts to develop an effective vaccine should consider soliciting HIV-specific mucosal IgA production. In support of this, mucosal IgA and IgG, elicited through mucosal vaccination with HIV-1 gp41 subunit virosomes in nonhuman primates, prevented systemic invasion following vaginal simian-HIV challenge by blocking transcytosis and by mediating antibody-dependent cellular cytotoxicity (ADCC) [39]. Thus, “control” of HIV disease progression is associated with normal mDC BLyS expression, likely contributing to “preservation” of the B cell compartment and to its capacity of generating both T-dependent and -independent effective B cell responses, such as mucosal IgA, viewed to block systemic invasion by the virus (Figure 1).

## Does DC Status Modulate the Outcome of CD4^+^ T Cell Effector/Target Availability for HIV?

DCs are involved in maintaining a balance between tolerance and protective immunity. This process is pivotal at mucosal sites, where the main battle with HIV takes place and immune homeostasis warrants peripheral integrity. Recent studies have demonstrated that the homeostatic balance of regulatory versus inflammatory responses at the mucosal level involves cross talk between epithelial cells and DCs [40]–[42]. Importantly, such mucosal immune homeostatic processes are thought to operate mainly through transforming growth factor (TGF)-β and retinoic acid (RA)-dependent mechanisms [40], modulating T regulatory/effector ratios as well as IgA production [31], [36]. Interestingly, TLR-mediated epithelial cell:DC cross talk at the level of human tonsillar crypts was shown to orchestrate B cell CSR through modulation of BLyS levels via thymic stromal lymphopoietin (TSLP) or secretory leukocyte protease inhibitor (SLPI) [41]. As depicted in Figure 1, the incapacity to keep a balance in homeostatic processes, which is likely to occur in individuals who progress with HIV infection, will promote inflammation and lead to disease perpetuation and excessive generation of T effectors, prime targets for HIV [43]. In contrast, the capacity to maintain immune homeostasis at mucosal sites may allow for better control of HIV infection. This view is consistent with a report showing that early prevention of macrophage inhibitory protein (MIP)-3α (CCL20) production in the genital tract of SIV-susceptible female macaques prevented excessive recruitment of DC populations, establishment of an inflammatory milieu, and infection, despite repeated intra-vaginal exposure to high doses of SIV [44]. Furthermore, studies of SIV infection in non-pathogenic animal models have shown that their control of disease progression appears linked to better management of the aberrant immune activation by early onset of anti-inflammatory IL-10 production and T regulatory activity. Moreover, fewer Th17 effector target cells were generated in non-pathogenic than in pathogenic SIV infections [43], a process linked to a low type I interferon (IFN)-gene profile and low TLR7-signalling [45]. Interestingly, both type I IFN- and TLR7-signalling are involved in the regulation of BLyS expression patterns by DCs [20], [46]. The fact that low concentrations of BLyS were shown to induce IL-10-producing murine splenic MZ “regulatory” B cells, whereas elevated BLyS concentrations promoted MZ B cell activation, suggests that BLyS may play an important role in modulating the outcome of T regulatory/effector balance via B cells [47].

Indeed, there is an increasing body of experimental evidence demonstrating the role of B cells in regulating the development, proliferation, and maintenance of CD4^+^ T cell populations, through both contact and/or cytokine mediated effector/regulatory functions [48], [49]. Sporadic depletion of B cells is an effective therapy for several T cell–mediated autoimmune diseases, allowing for a decline in inflammation and favouring the emergence of regulatory populations [48]. Decreased effector and increased regulatory CD4^+^ T cell functions were observed following blocking of BLyS in type I-diabetic (NOD) mice [50]. In a collagen-induced model of rheumatoid arthritis, BLyS over expression was shown to promote the expansion of Th17 cells, and BLyS gene silencing inhibited DC-mediated Th17 cell differentiation in vitro [51]. These observations suggest that DCs may influence T cell differentiation in a BLyS-mediated manner either directly and/or indirectly via modulation of B cell regulatory/effector functions.

The overall outcome of HIV disease may depend on the host's capacity to maintain dendritic cell (DC) homeostasis at mucosalsites, where DC populations, one of the earliest cell types to be exposed to the virus, present an inherent capacity to modulate the balance between tolerance and protection. DCs may influence mucosal B cell responses against HIV through contact and/or production of B cell growth factors such as B lymphocyte stimulator (BLyS/BAFF), which in turn modulate the outcome of CD4^+^ T cell HIV effectors/targets. Recent observations of HIV/SIV infections in non-pathogenic animal models and from mucosal vaccination of nonhuman primates suggest that maintenance of systemic integrity may be achieved through constraining highly efficient immune responses to mucosal sites.

## Concluding Remarks

BLyS expression levels correlate with both the extent to which the B cell compartment is compromised and HIV disease progression status. The fact that HIV elite controllers expressed relatively low levels of BLyS suggest that therapeutic blockage of BLyS in HIV progressors may restore balanced effector to regulatory cell ratios to reduce both HIV target cells and systemic immune activation that are the hallmarks of HIV disease progression.

## References

[1] Moir S, Fauci AS (2009). B cells in HIV infection and disease.. Nat Rev Immunol.

[2] Fontaine J, Chagon-Choquet J, Valcke HS, Poudrier J, Roger M (2011). High expression levels of B Lymphocyte Stimulator (BLyS) by dendritic cells correlate with HIV-related B cell disease progression in humans.. Blood.

[3] Jacobson MA, Khayam-Bashi H, Martin JN, Black D, Ng V (2002). Effect of long-term highly antiretroviral therapy in restoring HIV-induced abnormal B-lymphocyte function.. J Acquir Immune Defic Syndr.

[4] Calabrese LH, Kirchner E, Shrestha R (2005). Rheumatic complications of human immunodeficiency virus infection in the era of highly active antiretroviral therapy: emergence of a new syndrome of immune reconstitution and changing patterns of disease.. Semin Arthritis Rheum.

[5] Bekker V, Scherpbier H, Pajkrt D, Jurriaans S, Zaaijer H (2006). Persistent humoral immune defect in highly active antiretroviral therapy-treated children with HIV-1 infection: loss of specific antibodies against attenuated vaccine strains and natural viral infection.. Pediatrics.

[6] Batista FD, Harwood NE (2009). The who, how and where of antigen presentation to B cells.. Nat Rev Immunol.

[7] Macpherson G, Kushnir N, Wykes M (1999). Dendritic cells, B cells and the regulation of antibody synthesis.. Immunol Rev.

[8] Cerutti A, Rescigno M (2008). The biology of intestinal immunoglobulin A responses.. Immunity.

[9] Lekkerkerker AN, van Kooyk Y, Geijtenbeek TB (2006). Viral piracy: HIV-1 targets dendritic cells for transmission.. Curr HIV Res.

[10] Hanna Z, Kay DG, Rebai N, Guimond A, Jothy S (1998). Nef harbors a major determinant of pathogenicity for an AIDS-like disease induced by HIV-1 in transgenic mice.. Cell.

[11] Poudrier J, Weng X, Kay DG, Paré G, Calvo EL (2001). The AIDS disease of CD4C/HIV transgenic mice shows impaired germinal centers and auto-antibodies and develops in the absence of IFN-gamma and IL-6.. Immunity.

[12] Poudrier J, Weng X, Kay DG, Hanna Z, Jolicoeur P (2003). The AIDS-like disease of CD4C/human immunodeficiency virus transgenic mice is associated with accumulation of immature CD11bHi dendritic cells.. J Virol.

[13] Mackay F, Figgett WA, Saulep D, Lepage M, Hibbs ML (2010). B-cell stage and context-dependent requirements for survival signals from BAFF and the B-cell receptor.. Immunol Rev.

[14] Hässler S, Ramsey C, Karlsson MC, Larsson D, Herrmann B (2006). AIRE-deficient mice develop haematopoietic irregularities and marginal zone B-cell lymphoma.. Blood.

[15] Lindh E, Lind SM, Lindmark E, Hässler S, Perheentupa J (2008). AIRE regulates T-cell-independent B-cell responses through BAFF.. Proc Natl Acad Sci USA.

[16] Federico M, Percario Z, Olivetta E, Fiorucci G, Muratori C (2001). HIV-1 Nef activates STAT1 in human monocytes/macrophages through the release of soluble factors.. Blood.

[17] Mangino G, Percario ZA, Fiorucci G, Vaccari G, Manrique S (2007). In vitro treatment of human monocytes/macrophages with myristoylated recombinant NEF of human immunodeficiency virus type 1 leads to the activation of mitogen-activated protein kinases, IkappaB kinases, and interferon regulatory factor 3 and to the release of beta interferon.. J Virol.

[18] Quaranta MG, Tritarelli E, Giordani L, Viora M (2002). HIV-1 Nef induces dendritic cell differentiation: a possible mechanism of uninfected CD4 (+) T cell activation.. Exp Cell Res.

[19] He B, Qiao X, Klasse PJ, Chiu A, Chadburn A (2006). HIV-1 envelope triggers polyclonal Ig class switch recombination through a CD40-independent mechanism involving BAFF and C-type lectin receptors.. J Immunol.

[20] Yu H, Liu Y, Han J, Yang Z, Sheng W (2011). TLR7 regulates dendritic cell-dependent B cell responses through BLyS in immune thrombocytopenic purpura.. Eur J Haematol.

[21] Sabado RL, O'Brien M, Subedi A, Qin L, Hu N (2010). Evidence of dysregulation of dendritic cells in primary HIV infection.. Blood.

[22] Fontaine J, Coutlée F, Tremblay C, Routy JP, Poudrier J (2009). HIV infection affects blood myeloid dendritic cells after successful therapy and despite nonprogressing clinical disease.. J Infect Dis.

[23] Fontaine J, Poudrier J, Roger M (2011). Persistence of high blood levels of the chemokines CCL2, CCL19 and CCL20 during the course of HIV infection.. AIDS Res Hum Retroviruses.

[24] del Rio ML, Bernhardt G, Rodriguez-Barbosa JI, Förster R (2010). Development and functional specialization of CD103+ dendritic cells.. Immunol Rev.

[25] Weill JC, Weller S, Reynaud CA (2009). Human Marginal Zone B cells.. Annu Rev Immunol.

[26] Malaspina A, Moir S, Chaitt DG, Rehm CA, Kottilil S (2007). Idiopathic CD4+ T lymphocytopenia is associated with increases in immature/transitional B cells and serum levels of IL-7.. Blood.

[27] Pantaleo G, Graziosi C, Demarest JF, Butini L, Montroni M (1993). HIV infection is active and progressive in lymphoid tissue during the clinically latent stage of disease.. Nature.

[28] Varin MM, Le Pottier L, Youinou P, Saulep D, Mackay F (2010). B-cell tolerance breakdown in Sjögren's syndrome: focus on BAFF.. Autoimmun Rev.

[29] Malaspina A, Moir S, Ho J, Wang W, Howell ML (2006). Appearance of immature/transitional B cells in HIV-infected individuals with advanced disease: correlation with increased IL-7.. Proc Natl Acad Sci U S A.

[30] Cancro MP, D'Cruz DP, Khamashta MA (2009). The role of B Lymphocyte Stimulator (BLyS) in systemic lupus erythematosus.. J Clin Invest.

[31] Cerutti A (2008). The regulation of IgA class switching.. Nat Rev Immunol.

[32] Lesley R, Xu Y, Kalled SL, Hess DM, Schwab SR (2004). Reduced competitiveness of autoantigen-engaged B cells due to increased dependence on BAFF.. Immunity.

[33] Thien M, Phan TG, Gardam S, Amesbury M, Basten A (2004). Excess BAFF rescues self-reactive B cells from peripheral deletion and allows them to enter forbidden follicular and marginal zone niches.. Immunity.

[34] Stohl W, Cheema GS, Briggs WS, Xu D, Sosnovtseva S (2002). B lymphocyte stimulator protein-associated increase in circulating autoantibody levels may require CD4+ T cells: lessons from HIV-infected patients.. Clin Immunol.

[35] Rodriguez B, Valdez H, Freimuth W, Butler T, Asaad R (2003). Plasma levels of B-lymphocyte stimulator increase with HIV disease progression.. AIDS.

[36] Fagarasan S, Kawamoto S, Kanagawa O, Suzuki K (2010). Adaptive immune regulation in the gut: T cell-dependent and T cell-independent IgA synthesis.. Annu Rev Immunol.

[37] Shacklett BL (2010). Immune responses to HIV and SIV in mucosal tissues: ‘location, location, location’.. Curr Opin HIV AIDS.

[38] Tudor D, Derrien M, Diomede L, Drillet AS, Houimel M (2009). HIV-1 gp41-specific monoclonal mucosal IgAs derived from highly exposed but IgG-seronegative individuals block HIV-1 epithelial transcytosis and neutralize CD4(+) cell infection: an IgA gene and functional analysis.. Mucosal Immunol.

[39] Bomsel M, Tudor D, Drillet AS, Alfsen A, Ganor Y (2011). Immunization with HIV-1 gp41 subunit virosomes induces mucosal antibodies protecting nonhuman primates against vaginal SHIV challenges.. Immunity.

[40] Rescigno M, Disabatino A (2009). Dendritic cells in intestinal homeostasis and disease.. J Clin Invest.

[41] Xu W, He B, Chiu A, Chadburn A, Shan M (2007). Epithelial cells trigger frontline immunoglobulin class switching through a pathway regulated by the inhibitor SLPI.. Nature Immunol.

[42] Manicassamy S, Reizis B, Ravindran R, Nakaya H, Salazar-Gonzalez RM (2010). Activation of beta-catenin in dendritic cells regulates immunity versus tolerance in the intestine.. Science.

[43] Sodora DL, Allan JS, Apetrei C, Brenchley JM, Douek DC (2009). Toward an AIDS vaccine: lessons from natural simian immunodeficiency virus infections of African nonhuman primate hosts.. Nat Med.

[44] Li Q, Estes JD, Schlievert PM, Duan L, Brosnahan AJ (2009). Glycerol monolaurate prevents mucosal SIV transmission.. Nature.

[45] Mandl JN, Barry AP, Vanderford TH, Kozyr N, Chavan R (2008). Divergent TLR7 and TLR9 signalling and type I interferon production distinguish pathogenic and nonpathogenic AIDS virus infection.. Nat Med.

[46] Tezuka H, Abe Y, Asano J, Sato T, Liu J (2011). Prominent Role for Plasmacytoid Dendritic Cells in Mucosal T Cell-Independent IgA Induction.. Immunity.

[47] Yang M, Sun L, Wang S, Ko KH, Xu H (2010). Novel function of B cell-activating factor in the induction of IL-10-producing regulatory B cells.. J Immunol.

[48] Lund FE, Randall TD (2010). Effector and regulatory B cells: modulators of CD4+ T cell immunity.. Nat Rev Immunol.

[49] Gray D, Gray M (2010). What are regulatory B cells?. Eur J Immunol.

[50] Marino E, Villanueva J, Walters S, Liuwantara D, Mackay F (2009). CD4+ CD25+ T cells control autoimmunity in the absence of B cells.. Diabetes.

[51] Lai Kwan Lam Q, King Hung Ko O, Zheng BJ, Lu L (2008). Local BAFF gene silencing suppresses Th17-cell generation and ameliorates autoimmune arthritis.. Proc Natl Acad Sci U S A.

